# Impacts of Surgery on Symptom Burden and Quality of Life in Pituitary Tumor Patients in the Subacute Post-operative Period

**DOI:** 10.3389/fonc.2019.00299

**Published:** 2019-04-23

**Authors:** Mark R. Waddle, Mollie D. Oudenhoven, Casey V. Farin, Allison M. Deal, Riane Hoffman, Hojin Yang, Jennifer Peterson, Terri S. Armstrong, Matthew G. Ewend, Jing Wu

**Affiliations:** ^1^Department of Radiation Oncology Mayo Clinic, Jacksonville, FL, United States; ^2^Department of Pediatrics, University of Colorado, Denver, CO, United States; ^3^Department of Neurology, Duke University, Durham, NC, United States; ^4^Lineberger Comprehensive Cancer Center Biostatistics Core Facility, UNC, Chapel Hill, NC, United States; ^5^Department of Neurosurgery, University of North Carolina, Chapel Hill, NC, United States; ^6^Neuro-Oncology Branch, National Cancer Institute/National Institutes of Health, Bethesda, MD, United States

**Keywords:** pituitary, adenoma, surgery, QOL, subacute, post-operative

## Abstract

**Background:** Pituitary tumors are rare but are associated with significant symptoms that impact patients' quality of life (QOL). Surgery remains one of the most effective treatment options for long term disease control and symptom benefit, but symptom, and quality of life recovery in the subacute period has not been previously reported. This study aimed to better understand the impact of surgery on patients' symptom burden and QOL in the subacute post-surgical period.

**Methods:** Twenty-three adult patients with pituitary tumors undergoing surgical resection at University of North Carolina Cancer Hospital were enrolled in this study. M.D. Anderson Symptom Inventory Brain Tumor Module, European Organization for Research and Treatment of Cancer QLQ-C30 and QLQ-BN20 questionnaires were collected pre- and 1-month post- surgical resection and differences were analyzed for individual and groups of symptoms and QOL using Wilcoxon signed-rank tests.

**Results:** Twenty adult patients had both pre-operation and post-operation follow-up visits; 60% had functional pituitary adenomas. Seven symptoms including fatigue, memory, vision, numbness, speaking, appearance, and weakness were significantly improved at the 1-month post-operation visit while one symptom, sleep, worsened. Global Health Status/QOL measurements was improved minimally from 63 (SD 25) at pre-operation to 67 (SD 22) at 1-month post-operation without statistical significance.

**Conclusions:** This study demonstrated a rapid improvement of many symptoms in the subacute post-operative period in pituitary tumor patients. Disturbed sleep was identified as the only symptom to worsen post-operatively, encouraging potential prospective interventions to improve sleep, and subsequently improve the QOL in pituitary tumor patients following surgical intervention.

## Introduction

Pituitary tumors are relatively rare primary central nervous system (CNS) tumors in adults (1) but make up 10% of all neurosurgical interventions in the United States (2). They are often an incidental finding in autopsies and brain imaging studies (3). Although these tumors are usually benign, pituitary tumors have a significant impact on a patient's health and have been shown to cause cognitive dysfunction (4, 5), visual deficits (6–8), headaches (7), and an overall decline in quality of life (QOL) at the time of diagnosis (3, 8, 9). Multiple studies have demonstrated the adverse clinical effects of these tumors at presentation, yet there are little data to describe how these symptoms, and thus QOL, are impacted with treatment. Surgery, the primary definitive treatment for these tumors, carries risk of damage to surrounding structures such as the internal carotid artery and optic nerve, CSF leak, and/or hormone abnormalities (10). Van der Klaauw's group showed that patients with all subtypes of pituitary tumors experienced decreased QOL as far as 10–15 years after treatment when compared with healthy controls, but the added impact of surgery on these outcomes was not described (11, 12). Other studies have shown improved sino-nasal functioning in the long term, but have mixed results in the subacute period (13). Further, there is currently limited evidence for the impact of surgery on many other important quality of life metrics which may impact a patient's ability to function (13–15).

The burden of patient symptoms and QOL is an essential consideration and has been increasingly recognized in literature as a primary end point for both benign and malignant tumors (16–18). The World Health Organization defines QOL as “an individual's perception of their position in life in the context of culture and value systems in which they live and in relation to their goals, expectations, standards, and concerns” (7). The importance of the symptom and QOL endpoint in oncology care has resulted in a multitude of validated scales and surveys which are routinely used in research and in practice. The goal of this study is to characterize the common symptoms, side effects, and overall QOL of patients with pituitary tumors before and after surgical intervention in the subacute period with the use of validated QOL instruments. Additionally, we hope this study will help identify potential biologic underpinnings and guide early intervention, symptom screening following intervention, and to aid in general clinical management to further improve QOL in patients with pituitary tumors who undergo surgical resection.

## Materials and Methods

### Patient Population

This is a prospective study using validated questionnaires of symptom burden and QOL. The study was undertaken in accordance of Good Clinical Practice guidelines and the Declaration of Helsinki. All eligible patients were provided with IRB-approved consent forms and all participating patients provided written informed consent. Approvals for the study protocol and consent forms were obtained from the Institutional Review Board (IRB) at the University of North Carolina at Chapel Hill (UNC).

Eligible patients were 18 years old or above, English speaking, with adequate mental capacity to fill out questionnaire, and no other malignancy that required active anti-neoplastic treatment in the past 3 years. All patients had histological diagnosis of pituitary tumor at UNC from June 2011 to March 2014.

### Study Design

With UNC IRB approval, all eligible patients were provided with informed consent forms. Patients were given the questionnaires including M.D. Anderson Symptom Inventory Brain Tumor Module (MDASI-BT), European Organization for Research and Treatment of Cancer (EORTC) QLQ-C30 and QLQ-BN20, at their pre-operative clinic visit within 7 days prior to surgery. Longitudinal follow up was completed with the same pre-operative surveys given post-operatively at 1 month after surgery, which was defined as the “Sub-acute post-surgical period.” Patients without histological diagnosis of pituitary tumors were considered non-evaluable after the pathology reports were reviewed.

### Outcome Assessment

Three extensively validated symptom inventory questionnaires were selected to assess patient's symptom burden and QOL: the MDASI-BT, EORTC QLQ-C30, and EORTC QLQ-BN20. Baseline and one-month post-operative symptom burden and QOL scores were recorded for each questionnaire. Changes relative to baseline were calculated for each patient and those values were averaged and reported for symptom scores and QOL.

### MDASI-BT

MDASI-BT consists of 28 questions which can be completed in an average time of 5–10 min. It is composed of questions rated on an 11-point scale (0–10) to indicate the presence and severity of each symptom in the last 24 h, with 0 being “not present” and 10 being “as bad as you can imagine.” A total of 28 questions include 22 symptom questions (13 core symptoms, 9 brain-tumor-specific symptoms) (19, 20) and six interference with life questions, which are further divided into activity and mood-related items. The 22 symptom specific questions on MDASI-BT also measure six underlying constructs: (1) an affective factor comprised of distress, fatigue, sleep, sadness, and irritability, (2) a cognitive factor comprised of difficulty understanding, remembering, speaking and concentrating, (3) focal neurological deficits factor, including seizure, numbness, pain, and weakness, (4) treatment-related symptoms such as dry mouth, drowsiness, and appetite, (5) generalized disease status symptoms, including change in vision, change in appearance, change in bowel patterns, and shortness of breath, and (6) GI related factors, including nausea and vomiting (20). Symptoms on the MDASI-BT are those common in the brain tumor population as well as those associated with cancer therapies. The MDASI-BT has evidence of content and construct validity, discriminant validity by performance status and disease progression, and internal consistency (20).

### EORTC QLQ-C30 and QLQ-BN20

EORTC QLQ-C30 (Version 3.0) is composed of 30 questions organized into a global health status/QOL scale that include 5 functional scales (physical, role, emotional, cognitive, and social), 3 symptom scales (fatigue, nausea and vomiting, and pain), and a number of single items assessing additional difficulties (dyspnea, insomnia, appetite loss, constipation, diarrhea, and financial difficulties).

EORTC QLQ-Brain Cancer Module (EORTC QLQ-BN20) is a supplement to the EORTC QLQ-C30 specifically designed for brain tumor patients and consists of 20 questions scored as four multi-item scales (future uncertainty, visual disorder, motor dysfunction, and communication deficit) and seven single-item symptom scales (headaches, seizures, drowsiness, hair loss, itchy skin, weakness of legs, and bladder control).

The responses to both questionnaires were scored as outlined in the QLQ-C30 scoring manual to a score from 0–100 where a higher score represents a high/healthy level of functioning and a high quality of life, but a high level of symptomatology/problems, depending on the question (21, 22).

### Statistical Analysis

Descriptive statistics are provided for patient characteristics and questionnaire results. Wilcoxon signed-rank tests are used to evaluate if changes in symptom burden and QOL were significantly different compared to baseline for both the symptom burden and QOL, and unadjusted *p*-values are reported. All analysis was done using SAS software v9.3 (Cary, NC).

## Results

### Patient Characteristics

A total of 23 patients diagnosed with a pituitary tumor at UNC hospital from June 2011 to March 2014 completed pre-operative questionnaires. Of these, one subject withdrew, one did not have surgery, and one subject was lost to follow up. The remaining 20 subjects underwent first time surgery for a pituitary tumor and completed the 1 month follow up questionnaire in the subacute surgical period.

Age of all evaluable patients ranged from 24 to 77 years with a median age of 49 years and 50% were male. The baseline Karnofsky Performance Score (KPS) ranged from 80 to 90. Functional adenomas were most common representing 60% of all studied patients including 30% prolactinomas and 20% GH secreting adenomas (Table 1).

**Table 1:** Patient Characteristics for Studied Subjects.

**Characteristic**	**Category**	**Total (percentage)**
**Age**	<65 years	18 (90)
Mean: 51	>65 years	2 (10)
**GENDER**		
	Male	10 (50)
	Female	10 (50)
**RACE**		
	White	14 (70)
	Black	6 (30)
**BASELINE KPS**		
	80	12 (60)
	90	8 (40)
	100	0 (0)
**Cellular classification of pituitary tumor**	Non-functional adenoma	8 (40)
	Prolactinoma	6 (30)
	GH secreting pituitary adenoma	4 (20)
	Pituitary tumor secreting >1 hormone	2 (10)

*KPS, Karnofsky Performance Status; GH, Growth Hormone*.

### Symptom Burden Questionnaire Results

MDASI-BT symptom severity scores are shown in Table 2A. Pre-operative MDASI-BT questionnaire results showed the most severe symptom was fatigue, with mean (standard deviation) severity scores of 5.0 (SD 2.7). Seizure was the least severe symptom with mean scores of 0.2 (SD 0.5).

**Table 2A:** MDASI symptom severity score and standard deviation for each symptom at pre-operation and 1 month post-operation.

**How severe is your ______ at its worst (0-10)**	**Pre-op (*SD*)**	**One month Post-op (*SD*)**
Fatigue	**5.0 ± 2.7**	3.4 ± 2.4 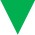
Pain	3.9 ± 3.5	3.0 ± 3.4
Impaired memory	3.7 ± 2.8	2.3 ± 2.1 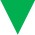
Drowsiness	3.6 ± 3.4	3.4 ± 3.0
Vision	3.4 ± 2.9	1.0 ± 1.0 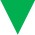
Disturbed sleep	3.1 ± 2.9	**4.8 ± 3.2** 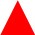
Numbness/tingling	3.1 ± 2.9	1.7 ± 2.9 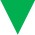
Feeling distressed	2.9 ± 3.3	3.0 ± 3.4
Irritability	2.7 ± 3.2	2.3 ± 3.1
Lack of appetite	2.3 ± 3.0	1.4 ± 2.5
Difficulty concentrating	2.2 ± 2.7	1.2 ± 1.3
Nausea	2.1 ± 3.0	0.6 ± 1.4
Shortness of breath	2.0 ± 2.4	1.1 ± 1.9
Dry mouth	1.9 ± 3.0	2.3 ± 2.7
Difficulty speaking	1.8 ± 2.4	0.8 ± 1.2 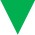
Appearance	1.8 ± 2.0	0.4 ± 1.0 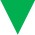
Sadness	1.6 ± 2.5	1.6 ± 2.6
Change in bowel pattern	1.5 ± 2.5	1.6 ± 2.0
Weakness on one side	1.4 ± 2.3	0.4 ± 1.0 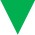
Difficulty understanding	0.8 ± 1.3	0.7 ± 1.2
Vomiting	0.8 ± 1.7	0.1 ± 0.2
Seizures	0.2 ± 0.5	0.4 ± 1.2
**HOW HAVE YOUR SYMPTOMS INTERFERED WITH: (0–10)**		
Work	**3.4 ± 3.8**	**2.5 ± 2.9**
General activity	3.2 ± 3.4	1.8 ± 2.6 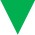
Enjoyment of life	2.8 ± 3.2	1.6 ± 2.7 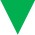
Mood	2.7 ± 3.0	2.2 ± 2.9
Walking	2.4 ± 3.3	1.0 ± 1.9 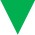
Relations	1.9 ± 2.7	1.6 ± 2.7

*Symptoms with a statistically significant change (p < 0.05) relative to pre-operative values are marked with an arrow, indicating the direction of change. (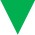
indicate less symptoms, 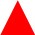
indicates more symptoms). The most severe symptoms for each time point are marked with bold text*.

**Figure 1:**
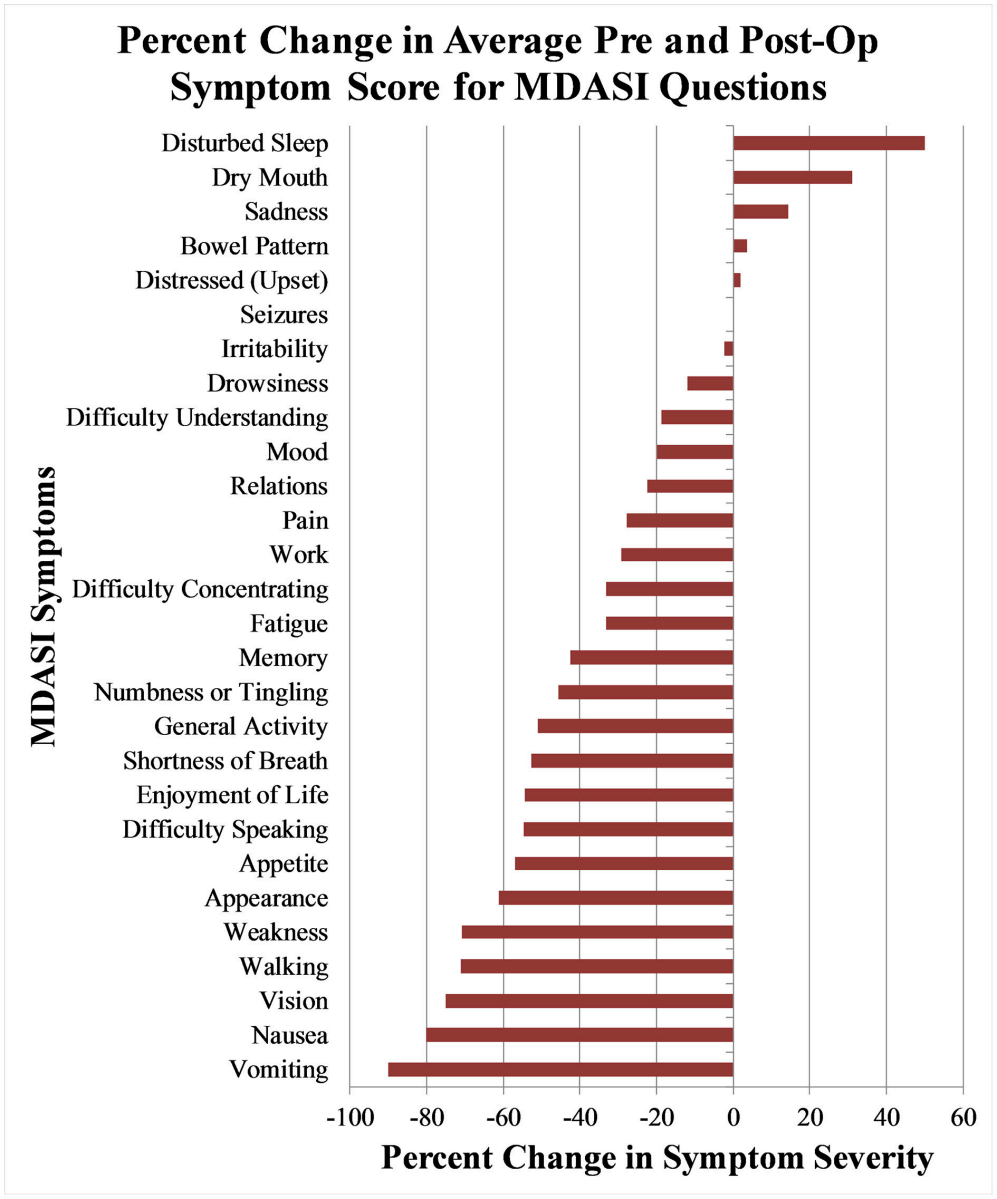
Percentage change of mean MDASI-BT symptom severity score between pre-operative questionnaire and 1 month post-operative questionnaires.

At 1-month post-operation, only one symptom, disturbed sleep, significantly worsened with a mean symptom severity score of 4.8 (*SD* 3.2, *p* = 0.03), a 55% increase from the baseline value of 3.5. Seizures also worsened by 166% from 0.15 (*SD* 0.5) to 0.4 (*SD* 1.2), and dry mouth slightly worsened from 1.9 (*SD* 3.0) to 2.3 (*SD* 2.7), both of which were small absolute changes and statistically non-significant. For the same time point, significant improvements were found in fatigue, impaired memory, vision, numbness/tingling, difficulty speaking, appearance, and weakness. The symptom that improved the most was vision, with a symptom severity score of 1.0 (*SD* 1.0), an improvement of 71% from the baseline. The percentage change of symptom severity score from baseline for all measured items can be seen in Figure 1.

The mean symptom severity scores for all symptoms pre-operation and 1-month post-operation were 2.3 (*SD* 1.2) and 1.7 (*SD* 1.2), respectively. In the interference with life category, three areas significantly improved from pre-operation to 1-month post-operation: general activity, enjoyment of life, and walking (*p* < 0.05).

**Figure 2:**
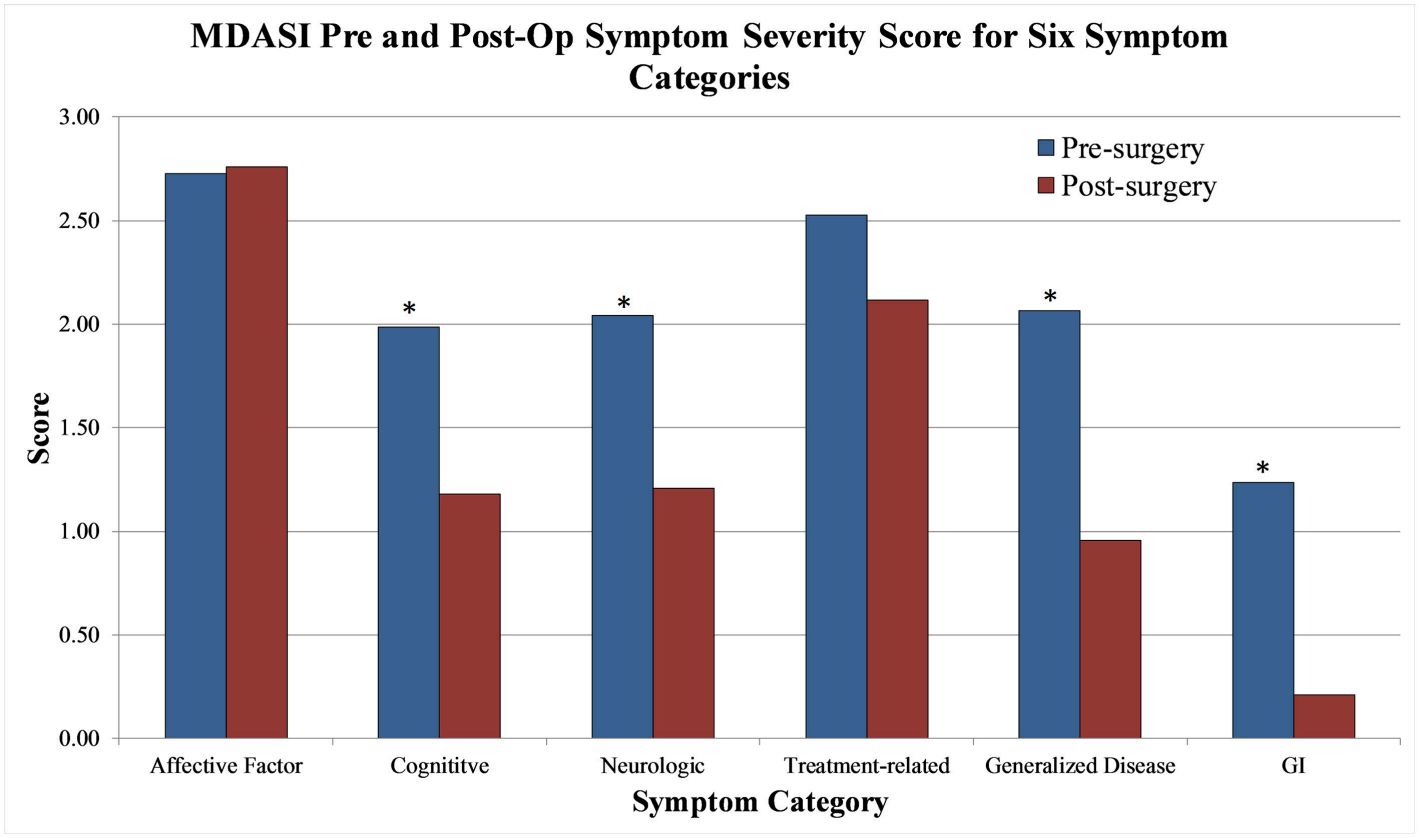
MDASI-BT symptom severity score pre-operative and 1 month post-op for six symptom categories.

Aggregate scores and their changes were calculated for the two MDASI-BT main categories and six MDASI-BT sub-categories, shown in Table 2B. Interference with life was significantly improved at 1-month post-operation, with a 43% improvement from the baseline. When the 22 symptom specific questions were analyzed in subgroups measuring six underlying constructs, four subgroups, including cognitive, neurologic, generalized disease, and gastrointestinal related factors were significantly improved by 41, 32, 55, and 79% from baseline, respectively, at 1-month post-operation follow-up (*p* < 0.05). Affective related factors did not show significant improvement at 1-month follow-up. The percent changes from baseline for the six subgroups are shown in Figure 2.

**Table 2B:** MDASI symptom severity main category and subcategory scores, and standard deviation for each symptom group at pre-operation and 1 month post-operation.

**How severe is your ______ at its worst (0–10)**	**Pre-op ±*SD***	**One month Post-op ±*SD***
**MDASI MAIN GROUPINGS (0–10)**		
Interference with Life	**3.0 ± 3.3**	1.7 ± 2.1 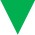
22 Symptoms (Core + Brain Tumor Specific)	2.4 ± 1.8	**1.7 ± 1.4**
**MDASI SUBCATEGORY GROUPINGS (0–10)**		
Affective factors	**3.0 ± 2.2**	**3.0 ± 2.5**
Treatment related	2.6 ± 2.4	2.3 ± 2.1
Cognitive	2.2 ± 2.0	1.3 ± 1.0 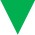
Neurologic	2.2 ± 1.7	1.5 ± 1.7 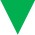
Generalized disease	2.2 ± 1.7	1.0 ± 1.0 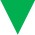
Gastrointestinal	1.4 ± 2.1	0.3 ± 0.7 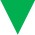

*Symptoms with a statistically significant change (p < 0.05) relative to pre-operative values are marked with an arrow indicating the direction of change. (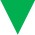
indicates less symptoms, 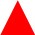
indicates more symptoms). The most severe symptoms for each time point are marked with bold text*.

### Quality of Life Results

EORTC QLQ-C30 and QLQ-BN20 questionnaire results are shown in Table 3. Pre-operatively, the most severe symptoms were headaches, drowsiness, fatigue, pain, and insomnia. Headaches were most severe with a symptom severity score of 53 (*SD* 35). The least severe was seizures with a symptom score of 0.

**Table 3:** EORTC QLQ-C30 and BN20 scaled scores and standard deviation for each symptom at pre-operation and 1 month post-operation.

**QLQ-C30 during the past week, have you had trouble with: (0–100)**	**Pre-op ±*SD***	**One month Post-op ±*SD***
Fatigue	**31 ± 24**	35 ± 30
Pain	**31 ± 27**	34 ± 36
Insomnia	30 ± 32	**40 ± 34**
Financial difficulties	28 ± 35	25 ± 36
Constipation	19 ± 30	18 ± 26
Dyspnea	17 ± 23	18 ± 28
Nausea and vomiting	13 ± 19	5 ± 15
Appetite loss	13 ± 17	12 ± 27
Diarrhea	8 ± 15	7 ± 17
**FUNCTIONAL SCALES (0–100)**		
Cognitive functioning	71 ± 29	79 ± 22 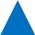
Role functioning	75 ± 29	73 ± 31
Emotional functioning	78 ± 24	77 ± 27
Physical functioning	82 ± 19	80 ± 25
Social functioning	83 ± 27	81 ± 32
Global health status/QoL (0–100)	**63 ± 25**	**67 ± 22**
**BN-20 during the past week, have you had trouble with: (0–100)**		
Headaches	**53 ± 35**	**47 ± 40**
Drowsiness	37 ± 29	25 ± 24 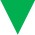
Visual disorder	24 ± 27	8 ± 13 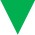
Itchy skin	23 ± 34	12 ± 20
Future uncertainty	21 ± 27	12 ± 17
Weakness of legs	20 ± 33	13 ± 23
Motor dysfunction	16 ± 23	16 ± 22
Communication deficit	16 ± 23	12 ± 20
Hair loss	12 ± 27	9 ± 24
Bladder control	10 ± 24	5 ± 16
Seizures	0 ± 0	2 ± 8

*Symptoms with a statistically significant change (p < 0.05) relative to pre-operative values are marked with an arrow indicating the direction of change. (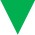
indicates less symptoms, 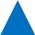
indicates improved functioning) The most severe symptoms and the lowest level of functioning for each time point are marked with bold text*.

By 1-month post-operation, the symptom that improved the most was visual disorders, which improved by 67%, while insomnia worsened the most, by 33%. Headaches remained the most severe symptom at 47 (*SD* 40). Statistically significant improvements were shown in cognitive functioning (*p* < 0.01) and visual disorders (*p* < 0.01) from pre-operation to 1-month post- operation.

Global status of health/QOL was stable from pre-operative baseline to 1-month post-operation with scores of 63 (*SD* 25) and 67 (*SD* 22), respectively, an improvement of 5%.

## Discussion

This study is consistent with prior studies and demonstrates that the majority of patients are symptomatic prior to intervention (11, 12). However, we successfully showed a novel finding that in this group of otherwise healthy individuals, many symptoms improve rapidly with surgical intervention, as outlined in the result section above.

The initial improvement in vision was expected. Vision loss is a well-known symptom of pituitary tumors due to compression of the overlying optic chiasm and nerves which improves rapidly following surgical decompression and removal of the tumor (6). However, many other symptoms including fatigue, memory, appearance, difficulty speaking, numbness, and appetite showed improvement in the subacute post-operative period in this study, which is a finding with little to no prior data for comparison in this patient population.

Two interference items, walking and enjoyment of life also improved. In fact, the interference with life category and four of six subcategories for symptom burden in MDASI-BT (cognitive, neurologic, generalized disease, and gastrointestinal) showed statistically significant improvement at 1-month post-operation, as shown in Table 2B, demonstrating a multitude of improvements in symptom burden and quality of life. These findings may be useful in counseling patients' suffering from these symptoms or clinicians looking to weigh the risk/benefit ratio of surgery for a given patient.

Patients' functioning improved as well. Self-reported measures of cognitive functioning improved significantly from pre-operation to 1-month post-operation, while role functioning, emotional functioning, physical functioning and social functioning remained the same.

Our findings are unique. A prior study by Glicksman et al. showed that at 3 months post-operatively there was a significant improvement of sino-nasal symptoms per the Sinonasal Outcome Test (SNOT-22), which demonstrated improvements in Rhinologic, Extranasal, Ear/facial, Psychological, and Sleep domains, that continued to improve over a 2 year period (23). However, this study failed to report on either acute or subacute changes in QOL. Another study by McCoul et al. (13) reported generalized symptom burden at 3 weeks and showed transient overall worsening of symptoms at this time point, driven largely by site specific factors such as intranasal edema, crusting, and hyposmia, but reported minimal to no improvement in other quality of life endpoints until 6–12 weeks post-operatively (13). However, these studies were both limited by the quality of life metrics they reported on and did not test for symptoms which showed dramatic improvements in our study, such as memory, appearance, difficulty speaking, numbness, appetite, cognitive function, walking, and enjoyment of life. Therefore, our findings are encouraging that, despite a possible worsening of surgery related symptoms in the subacute surgical period after sino-nasal resection shown in prior studies, many symptoms that can have a dramatic impact on a patient's QOL did improve rapidly. Additionally, we found that the MDASI-BT in this patient population was an excellent test to accurately define quality of life changes which are pertinent and important. The MDASI-BT has evidence of content and construct validity, discriminant validity by performance status and disease progression, and internal consistency. For this reason, we encourage its use for patients undergoing treatment of pituitary adenomas. However, more extensive research and experiences of evaluating pituitary patients with current QOL questionnaires may lead to a pituitary specific QOL instrument, which could better serve this patient population.

One symptom, disturbed sleep, did show worsening in the subacute period 1-month post-operation and was the most severe symptom following surgery in our studied patients. Disturbed sleep increased from a low range severity before surgery to nearly high range severity after surgery, an increase of 55%. Similarly, insomnia increased in severity by 33% in the EORTC-C30 questionnaire testing, but without statistical significance. Impairments in sleep have been previously described in patients with cancer, and may correlate with decreased total and free cortisol levels, due to a disrupted hypothalamus-pituitary-adrenal (HPA) axis (24). Our patients had similar sleep impairments, and our findings suggest that pituitary surgery for pituitary tumors may temporarily further disrupt the HPA axis, possibly worsening an already impaired sleep-wake cycle. Alternatively, the patient's daily routine may be temporarily altered leading to poor sleep hygiene, and the patients may be on treatment such as steroids which are known to have side effects such as disturbed sleep. Regardless of the underlying cause, disturbed sleep is likely a symptom that could benefit from routine screening possibly with sleep studies, avoidance of steroids if possible, and/or prophylactic treatment post-operatively, and prior studies are encouraging in that this symptom significantly improves by 6 months to 12 months post-operatively (23).

Another troubling symptom on the secondary questionnaire testing was headaches, which was the most severe symptom reported in both pre-operative and post-operative surveys. This symptom marginally improved from baseline with an improvement of 11% at 1-month post-operation, although not statistically significant. While this is a symptom well known to clinicians treating pituitary tumors, the severity and lack of change with treatment indicate that, on average, patients are experiencing significant distress from this symptom. Continued attention should be given to symptomatic relief of headaches even after resection, as surgical treatment is not expected to dramatically improve this problem, at least in this studied population.

This study has several limitations. The primary limitations, and ones common to studies of pituitary tumors, was that the sample size obtained was not large and thus only powered to detect large differences, and that limited follow-up data is available to assess for further longitudinal effects. Although the study took place at a large academic hospital with a robust pituitary program, the number of patients undergoing first time pituitary surgery was not as large as predicted, and there were several gaps in patient accrual. Additionally, several patients were excluded for not speaking English, as described in the inclusion criteria. As a result, there were 20 patients followed to 1-month post-operation. While a size of 20 subjects was adequate to find statistical significance in very large changes, it is likely that many more subtle changes were not identified. As an example, sleep disturbance was found to have a statistically significant worsening after surgery on the MDASI-BT, whereas insomnia was worse, but not statistically significant on EORTC QLQ-C30. This is likely a matter of sample size resulting in two validated questionnaires yielding similar results, yet only one reaching statistical significance. Future studies could be improved by collaboration with multiple other institutions to increase accrual and by having questionnaires and consent forms available in multiple languages.

Finally, we were unable to conduct a sub-group analysis between pituitary tumor types, hormone status, or patient demographics. Studies have shown symptoms vary between pituitary tumor groups and hormone status. For example, appearance score has been found to be the worst in patients with acromegaly, and patients with Cushing disease secondary to pituitary adenomas have the most impaired QOL (7, 12). Sub-group analysis would allow physicians to identify certain patient groups with more severe symptoms to better target symptom control or prevention.

Despite the above limitations, we believe that this study has important clinical implications. This study successfully prospectively characterizes specific symptoms which have previously not been investigated in pituitary adenomas. A strength of this study is that, to our knowledge, it is the first prospective study examining symptom burden in a number of measures following pituitary tumor surgery. Additionally, the use of comprehensive and very well validated questionnaires, MDASI-BT, EORTC-C30, and EORTC-BN20, allowed a complete assessment of symptoms in this disease group. We successfully identified sleep disturbance and headaches as symptoms that can be targeted clinically either with improved monitoring, pre-emptive treatment, and/or prospective future study. The study results also identified several other symptoms which significantly improved after the surgery. Future interventional studies may include multi-centered collaboration with well-established research infrastructure for the investigation of symptom burden and health related quality of life. We hope these data will aid clinicians both in pre-operative and post-operative patient counseling, provide clinicians with improved awareness of troublesome symptoms associated with surgery, and continue the progress being made in the quality of life of brain tumor patients.

## Ethics Statement

The study was undertaken in accordance of Good Clinical Practice guidelines and the Declaration of Helsinki. All patients provided their written informed consent. Approvals for the study protocol and consent forms were obtained from the Institutional Review Board (IRB) at the University of North Carolina at Chapel Hill (UNC). Eligible patients were of or above 18 years old.

## Author Contributions

JW, TA, ME, CF, MO, JP, and MW contributed to the design and implementation of the research, to the analysis of the results and to the writing of the manuscript. AD and HY contributed to the statistical analysis. RH contributed to design and implementation of the research, to the analysis of the results and to the writing of the manuscript.

### Conflict of Interest Statement

The authors declare that the research was conducted in the absence of any commercial or financial relationships that could be construed as a potential conflict of interest.
